# Genome and pan-genome analysis to classify emerging bacteria

**DOI:** 10.1186/s13062-019-0234-0

**Published:** 2019-02-26

**Authors:** Aurélia Caputo, Pierre-Edouard Fournier, Didier Raoult

**Affiliations:** 1 Aix Marseille Univ, IRD, APHM, MEPHI, IHU-Méditerranée Infection, Marseille, France; 2 Aix Marseille Univ, IRD, APHM, SSA, VITROME, IHU-Méditerranée Infection, Marseille, France

**Keywords:** Genomic, Novel species, Pan-genome, Taxonomy

## Abstract

**Background:**

In the recent years, genomic and pan-genomic studies have become increasingly important. Culturomics allows to study human microbiota through the use of different culture conditions, coupled with a method of rapid identification by MALDI-TOF, or 16S rRNA. Bacterial taxonomy is undergoing many changes as a consequence. With the help of pan-genomic analyses, species can be redefined, and new species definitions generated.

**Results:**

Genomics, coupled with culturomics, has led to the discovery of many novel bacterial species or genera, including *Akkermansia muciniphila* and *Microvirga massiliensis*. Using the genome to define species has been applied within the genus *Klebsiella*. A discontinuity or an abrupt break in the core/pan-genome ratio can uncover novel species.

**Conclusions:**

Applying genomic and pan-genomic analyses to the reclassification of other bacterial species or genera will be important in the future of medical microbiology. The pan-genome is one of many new innovative tools in bacterial taxonomy.

**Reviewers:**

This article was reviewed by William Martin, Eric Bapteste and James Mcinerney.

**Open peer review:**

Reviewed by William Martin, Eric Bapteste and James Mcinerney.

## Background

The study of digestive bacterial ecosystems was initially explored by microbial culture in the 1970s [1–4]. The birth of genomics, followed by the development of Next Generation Sequencing (NGS) methods in 2004, made it possible to discover the uncultivable, such as *Akkermansia muciniphila* [5] thanks to metagenomics. Since the emergence of metagenomics, microbial culture has gradually been replaced by molecular tools for complex microbiota study [6]. In 2015, a new approach called “culturomics” was developed and intensive culture assays were carried out to select the 18 best culture conditions to cultivate the largest number of isolates [7]. Culturomics has also allowed the identification of a large number of prokaryotic species, as it allows the simultaneous combination of different culture conditions, using 16S rRNA gene amplification and matrix-assisted laser desorption/ionization time-of-flight (MALDI-TOF) [8]. As a result, by increasing the number of species of bacteria being discovered, a new polyphasic method for bacterial species description has emerged; the “taxonogenomics” [9]. In order to describe a new bacterium, taxonogenomics complements classic features with the description of the whole genome sequence with the proteomic information obtained by MALDI-TOF MS and has permitted to describe, for example, *Microvirga massiliensis* [10].

**Fig. 1:**
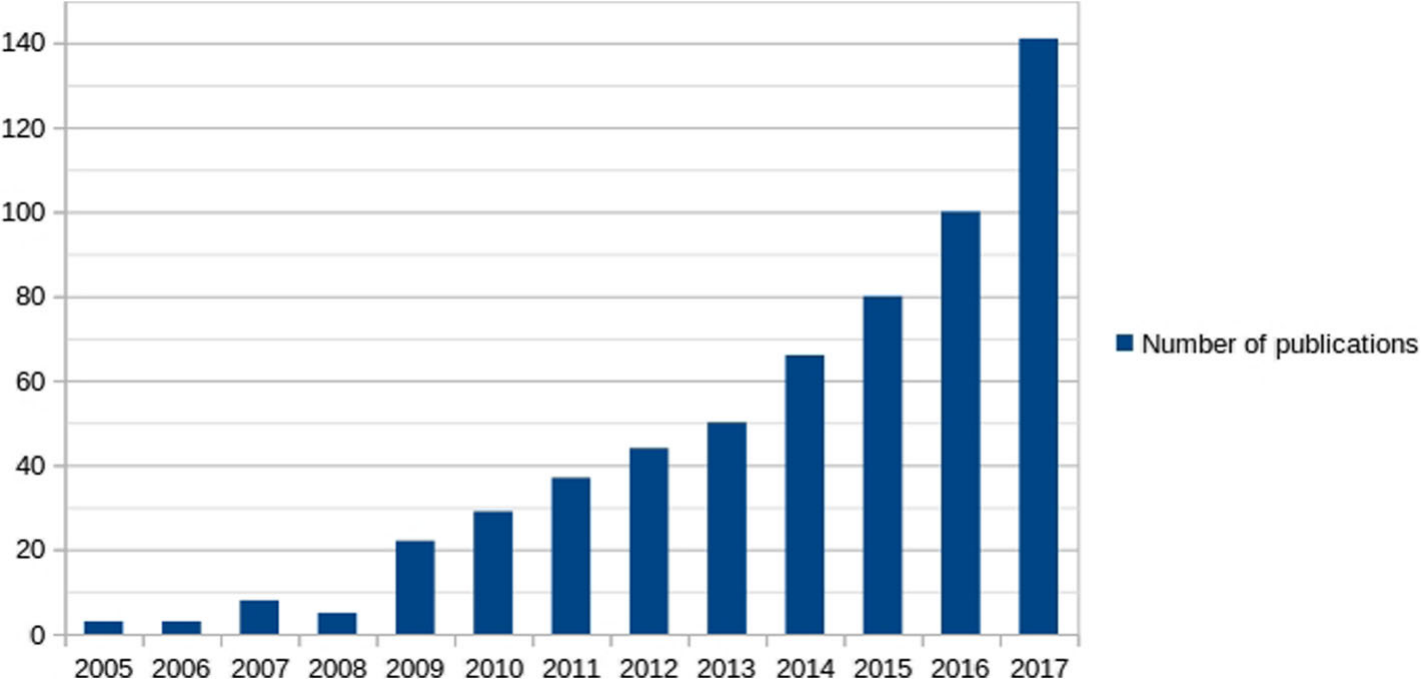
Number of publications per year for all pan-genome studies in the genomic database on the PubMed website (https://www.ncbi.nlm.nih.gov/pubmed)

The current classification of bacterial species relies on a combination of phenotypic and genotypic properties [11–13]. The genotypic criteria used for bacterial taxonomy was the genomic G + C content composition, DNA-DNA hybridization, and, later, the 16S rRNA gene [14, 15]. However, these genotypic criteria were limited since they required the use of restrictive genetic tools. For instance, DNA-DNA hybridization uses a 70% threshold for species discrimination. However, it cannot be used for all prokaryote genera, as described for *Rickettsia* species [16, 17]. Furthermore, the comparison of the single gene 16S rRNA [18–20], as well as the low conventional divergence between two 16S rRNA genes [21] of two organisms, causes a slight and limited bacterial description [22]. Indeed, the experiments of Acinas et al. involving 76 whole genomes shows an extreme diversity (11.6%) of the identity of 16S rRNA genes in the bacteria *Thermoanaerobacter tengcongensis* [23]. Except for *Thermoanaerobacter tengcongensis*, 16S rRNA gene is generally more conserved, and therefore not universally reliable for determining taxonomic relationships at the species level. Moreover, the variation of nucleotides observed in several copies of rRNA genes in single organisms, as well as the possibility that 16S rRNA genes are derived from horizontal gene transfer, can lead to well-established relationships between taxa, into phylogenetic trees [18]. Nevertheless, 16S rRNA gene is currently the gold standard tool for prokaryotic taxonomy [13]. With the emergence of first-generation sequencing in 1975–77 [24, 25], followed by high-throughput sequencing in 2004 [26], access to complete genetic information was deeply revolutionized. Thanks to these modern high-throughput sequencing technologies, a considerable amount of data is generated, enabling studies based on pan-genomic analyses (Fig. 1). The first definition of the pan-genome was proposed by Tettelin et al. [27] in 2005, just after the beginning of the era of high-throughput sequencing. A pan-genome can be defined as being the entire gene content belonging to a study group [28–30]. The applications are multiple, including the study of pathogenicity [31, 32], the mobilome [33], resistome [34], prediction of the lifestyle of bacteria [35], and also for taxonomy. Indeed, pan-genome study allows a reclassification of the species [36], thus clarifying and improving the traditional criteria previously presented.

## Mapping strategy

### NGS application in genomics and metagenomics

This technical revolution offers new fields of application, such as genomics (whole-genome sequencing, WGS), single-cell sequencing (SCS) and metagenomics. In particular, complete genome sequencing allows a better understanding of the genetic basis of phenotypic variability, but also to analyze biodiversity and estimate the diversity of single nucleotide polymorphism (SNP) [37]. Single-cell genomics allows the sequencing of a single isolated cell by capturing and amplifying its DNA. Single-cell sequencing is able to produce partial [38] and complete [39] genomes. SCS DNA allows the identification and assembly of genomes from uncultivated microorganisms [40–43] but it is estimated that only 1% of bacterial species have been cultivated in laboratory [44]. Nevertheless, SCS has its own limits along with inherent biases [40, 43], especially during the amplification step, including false-positive and false-negative errors, allelic dropout events and coverage non-uniformity [45]. However, single-cell genomics and metagenomics are two complementary approaches to analyze bacterial communities [46]. The sequencing of metagenomes has made it possible to inventory the diversity of microbial ecosystems [47] and to determine the interactions between microorganisms in ecosystems [48]. This technique, which allows DNA sequencing of bacteria present in specific environments, offers many advantages. Indeed, it opens a window on a world of great wealth that was hitherto totally unknown.

### Assembly, finishing and annotations

Organism sequencing has recently become accessible in many laboratories where specific consortium sequencing projects are proposed, and currently the number of projects is exponentially growing. However, NGS technologies have higher error rates (~ 0.1–15%) and smaller read lengths (35–1000 bp) than those obtained from Sanger sequencing platforms [49]. After sequencing, the data produced (reads) are computationally reconstructed into longer continuous sequences (contigs), a step called assembly [50]. This process consists of an overlap of reads aimed at reconstructing the initial sequence of the genome. It’s called de novo when no reference is available. The assembly with Sanger data is based on two-by-two comparison of reads, looking for overlapping sequences of minimal length with associated identity percentages (CAP assembler, for example [51]). The change of scale due to the huge volume of data, the short-read lengths and the non-uniform confidence in base calling, excluded this assembly strategy. The most commonly used approach for the assembly of short-read data is based on treating k-mers. All k-mers length substrings of all sequencing reads appear as nodes in a graph. The nodes are joined by edges if the nodes share a k-1 length substring. This graph is known as the de Bruijn graph [52]. Short read de Bruijn graph assemblers determine optimal paths through this graph to form the contigs of an assembly. Each contig ends when there is no outgoing edge from a visited node, or when the branching in the graph becomes too complex to be resolved. Frequently, the raw de Bruijn graph is reduced by collapsing any linear chains of nodes and edges into a single metanode. An assembly quality can then be assessed using a set of metrics. The usual ones are the total count of contigs, scaffolds, their total length, N50 (length of the smallest contig in the set of contigs that represent at least 50% of the genome), and their average length [53, 54]. Bilen et al., removed scaffolds less than 800 bp in size or less than 25% of the median depth (identified as possible contaminants) [55]. A good metric is also the proportion of reads mapped back, or not, to the contigs [53]. The assembly quality is an important criterion for ulterior analysis such as rate of lateral transfer or annotation [56]. The next important step required is genome annotation. To make genomic data valuable, a reliable and correct annotation is essential [57]. It is used to identify, locate and distinguish gene function using similarities when studying protein databases by BLAST [58], and can provide a basis for many genome analyses [59]. Organisms whose genome is now fully sequenced have revealed that nearly 40% of the genes identified have no assigned function; either because they do not resemble any known genes, or because (for half of them) they resemble other genes with unknown function [60]. The first step in annotation is to predict gene function, which is usually done individually for each gene using computational tools. However, the identification of gene function requires the combination of several complementary experimental approaches, whether by computer (in silico analysis), biochemical, or genetic (in vivo and in vitro analysis). The second step in annotation is to identify relationships between genes, proteins and regulatory elements. These relationships can be of very varied nature: physical interactions between proteins/DNA, proteins/RNA and proteins/proteins, networks regulating gene expression, metabolic pathways or others.

### Example of a mapping study: *Akkermansia muciniphila*

Powerful approaches based on mapping short-read sequences to a reference genome are used to analyze WGS data from closely related isolates [5, 61–64]. Several bioinformatics tools are used in a mapping study, such as the CLC genomics Workbench (CLC bio, Aarhus, Denmark).

By using metagenomics data from a human stool sample, the genome of *Akkermansia muciniphila* was successfully assembled after mapping the short-read sequences [5]. The stool sample was from a patient admitted to the intensive care unit and treated with a 10-day course of imipenem [65]. High-level colonization by the *Verrucomicrobia* phylum was reported in this patient. Indeed, the reads from pyrosequencing are classified in the phylum range and up to 84% of these reads belong to *Verrucomicrobia* phyla. Reads were generated from a SOLiD sequencer, whereas short-reads shotgun and paired-end runs were generated on a 454 sequencer. Both technologies generated 1.4 G bases of metagenomic sequence data from the sample. The several mapping sequences from SOLiD and 454 data against the *A. muciniphila* type strain ATCC BAA-835 allowed to obtain the genome of *A. muciniphila* strain *Urmite* with 1 scaffold and 58 contigs.

The presence of a range of putative antibiotic resistance genes (ARGs) from different antibiotic classes has been demonstrated in a recent study of *A. muciniphila* strain *Urmite* [5]. The putative ARG were beta-lactamase, macrolides, vancomycin, chloramphenicol, sulfonamide, tetracycline and trimethoprim.

The short-read sequence mapping approach of a reference genome has been successful in assembling a genome directly from a human stool sample. However, this approach remains limited since very divergent sequences, not present in the reference, could not be detected [64]. Indeed, if the draft genome obtain contains highly divergent additional genes with respect to the reference genome, we may lose this information and not be able to completely reconstruct the original genetic content. Consequently, mapping method cannot be used on very divergent genomes [5]. These limitations will be progressively lessened by the exponential growth of data and subsequent genomes available in generalist databases.

## Finding novel species

### Novel species identification

#### - Culturomics (MALDI-TOF-MS and 16S rRNA)

In recent years, with the introduction of a rapid and inexpensive identification method using MALDI-TOF mass spectrometry, the volume of microbial culture has considerably increased, which now allows to more readily detect pathogenic bacterial species. This technique of reference for bacterial identification in clinical microbiology laboratories has also developed a new concept to study human microbiota also called “microbial culturomics” [7, 66]. Culturomics was developed in a study of a complex microbiota and allows a large number of isolates to be grown through the selection of the 18 best culture conditions [7, 9]. This technique is based on the diversification of culture conditions by varying the time and temperature of incubation but also culture medium composition and atmosphere [66]. In a preliminary work, this approach allowed the cultivation of 340 bacterial species, including 31 novel species, as well as species belonging to rare phyla (Synergistetes and Deinococcus-Thermus), using 212 different cultivation conditions [66]. A detailed analysis made it possible to select successively the 70 then the 18 most appropriate culture conditions in order to explore the greatest possible diversity for each sample. Another work has permitted to cultivated more than 50% of the known species of the human digestive tract, including 247 novel species [7, 8]. Novel species are identified by MALDI-TOF, and 16S rRNA gene sequencing for non-identified spectra in MALDI-TOF.

#### - other methods

Novel species were also identified with multilocus sequence analysis (MLSA) of several concatenated housekeeping gene sequences (*rrs*, *recA*, *gyrB*, *dnaK, glnII* and *rpoD*) [67, 68]. The housekeeping genes are usually involved in the expression and maintenance of genetic information at the transcription or translation level. The *recA* gene is essential for the maintenance and repair of DNA and is a good resolutive tool for predicting lineage and genus among rhizobial strains [69]. Phylogenetic analysis to discover new species also uses DNA sequences of the internal transcribed spacer 2 (ITS2) region [70].

The nucleotide sequence or the peptide sequence can also be used. In general, the peptide sequence is preferred, since it can avoid some biases inherent to the G + C content of the organism studied, as well as the degeneration on the third nucleotide of the codon [71].

### Taxonogenomics strategy and the example of a novel species study: *Microvirga massiliensis*

This new polyphasic strategy, called “taxonogenomics”, systematically combines phenotypic and genomic criteria [9, 72]. Using this strategy, 15 novel bacteria have officially been considered as new species and/or new genera in official validation lists No. 153 and No. 155 by the International Taxonomy Committee of the International Journal of Systematic and Evolutionary Microbiology [66, 73–83]. Lagier et al., identified a novel bacterial species, *Microvirga massiliensis*, from a human stool sample using culturomics and metagenomics approaches [66]. Recently, the description of the genome of *Microvirga massiliensis* sp. nov. strain JC119T was realised via the taxonogenomics approach [10]. The draft genome of this bacterium is 9,207,211 bp long, which is the largest bacterial genome of a human isolate. Of the 8762 predicted genes, 8685 were protein-coding genes, 77 were rRNA genes, including 21 rRNA genes, and the genome exhibits a G + C content of 63.28%.

## The bacterial pan-genome

### History of taxonomy and the concept of bacterial species

**Fig. 2:**
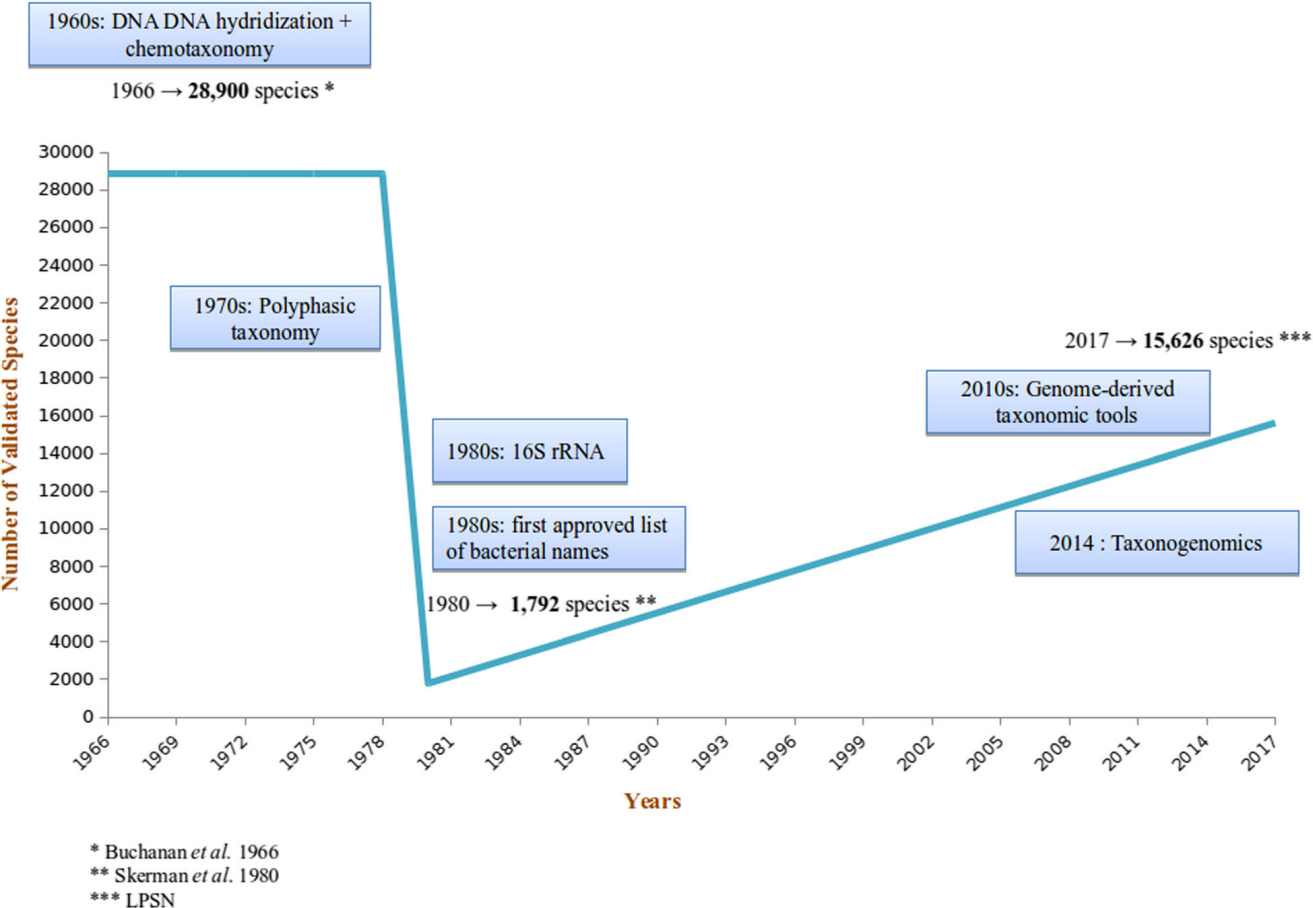
Validated number of bacterial names over the years. The blue box indicates the major advances in molecular biology

The number of bacterial species has constantly changed according to the classification criteria used. Indeed, in 1966, Buchanan et al. [84] published a census of 28,900 bacterial species, manifesting an urgent need for a better classification. The situation was improved in 1980 by Skerman, McGowan and Sneath [85], who, thanks to long and tedious efforts, managed to reduce the number of valid bacterial species to 1792. As an example, the taxonomy of *Salmonella* species was thoroughly modified, with the creation of subspecies and serovars instead of sub-genera and species, respectively, and the distribution of strains into two distinct species: *Salmonella enterica* and *Salmonella bongori*, on the basis of DNA-DNA hybridization [86, 87]. To date (May, 2017), this number has risen up to 15,626 species (listed in List of Prokaryotic names with Standing in Nomenclature (LPSN), www.bacterio.net) (Fig. 2).

Since the early 1990s, because of advances in molecular biology, bacterial classification has been in perpetual upheaval. Taxonomic information is essential regarding the identification, nomenclature and classification of microbial strains [88] and to better understanding the biodiversity and relationships among living microorganisms [89].

Currently, prokaryote taxonomy relies on polyphasic combinations of phenotypic properties (pathogenesis, morphology, environmental and culture conditions), chemotaxonomic properties (chemical composition of cellular components) and genotypic properties [11, 90], including DNA-DNA hybridization (DDH), DNA G + C content [91] and 16S rRNA sequence similarity [12, 92] The application of molecular hybridization methods provides a genomic definition of the bacterial species, taking into account the similarity rate and the thermal stability of the hybrids obtained by the DNAs of two bacterial isolates. Isolates belonging to the same species are characterized by homologies of their DNA, which result in hybridization percentages greater than or equal to 70%, and stability of the hybrids formed below 5 °C [93–95]. This definition is still recognized by the international bacterial taxonomy committees.

The development of DNA sequencing has led to the determination of a threshold in order to define the species based on the similarity of gene sequences, initially based on the 16S rRNA gene. These data were later compared to those obtained by DDH [96, 97]. As for the classification of prokaryotes, the 16S rRNA gene is an effective molecular marker due to its functional stability, conservation and universal presence [98]. However, for bacterial taxonomy, this gene has several limitations; notably, the presence of SNPs in the rRNA operon in a single genome [23, 99]; the use of a single gene that may not reflect the evolution history of the genome (~ 0.07% of a genome) [19, 100] or the high degree of conservation in a same genus, like *Brucella* or *Rickettsia* [101]. The divergence of 1.3% accepted from two sequences, corresponding to 50 million years of divergence [17, 22] also brings a limitation such that the presence in multiple of the 16 s rRNA genes and sometimes variable copies [102, 103]. VanBerkum et al., showed that a small portion of the 16S rRNA gene sequence of *Bradyrhizobium elkanii* is originated from a *Mesorhizobium* spp. genome by lateral transfer [100, 104].

What happens when two species have a 98.6% similarity percentage? Are they two distinct species? Establishing a threshold is not biological and cannot be based essentially on this criterion, especially when different thresholds are used by different biologists.

With the advent of whole genomes sequencing, phylogeny has entered a new era: the era of phylogenomics. Many studies have already demonstrated the importance of genomes in bacterial taxonomy by suggesting a focus on the presence or absence of genes within genomes [105–107]; the gene content [108]; the presence of SNPs or indels in conserved genes [109]; the comparison of orthologous genes [110]; the study of metabolic pathways and chromosome gene order [111, 112]; or by sequence similarity at the genome level, estimated by parameters such as “digital DDH”, Average Nucleotide Identity (ANI) or AGIOS using the Genome-To-Genome Distance Calculator (GGDC), ANI calculator and in-lab pipeline named Marseille Average Genomic identity (MAGi) softwares [113–116].

### Pan-genome study for taxonomic purposes: The *Klebsiella* genus

The taxonomic classification of *Klebsiella* species has been the subject of a long controversy. *Klebsiella* species are part of the large *Enterobacteriaceae* family, which are Gram-negative bacteria. Originally, the *Klebsiella* genus was divided into pathovars linked to the diseases they caused: *Klebsiella pneumoniae, Klebsiella ozaenae* and *Klebsiella rhinoscleromatis* [117]. With the development of new tools such as G + C content composition, DNA-DNA hybridization and 16S rRNA sequencing [11, 95], classification of *Klebsiella* species has been continuously revised [118, 119]. *K. ozaenae* and *K. rhinoscleromatis* were notably reclassified as *K. pneumoniae* subspecies [70, 120, 121].

**Fig. 3:**
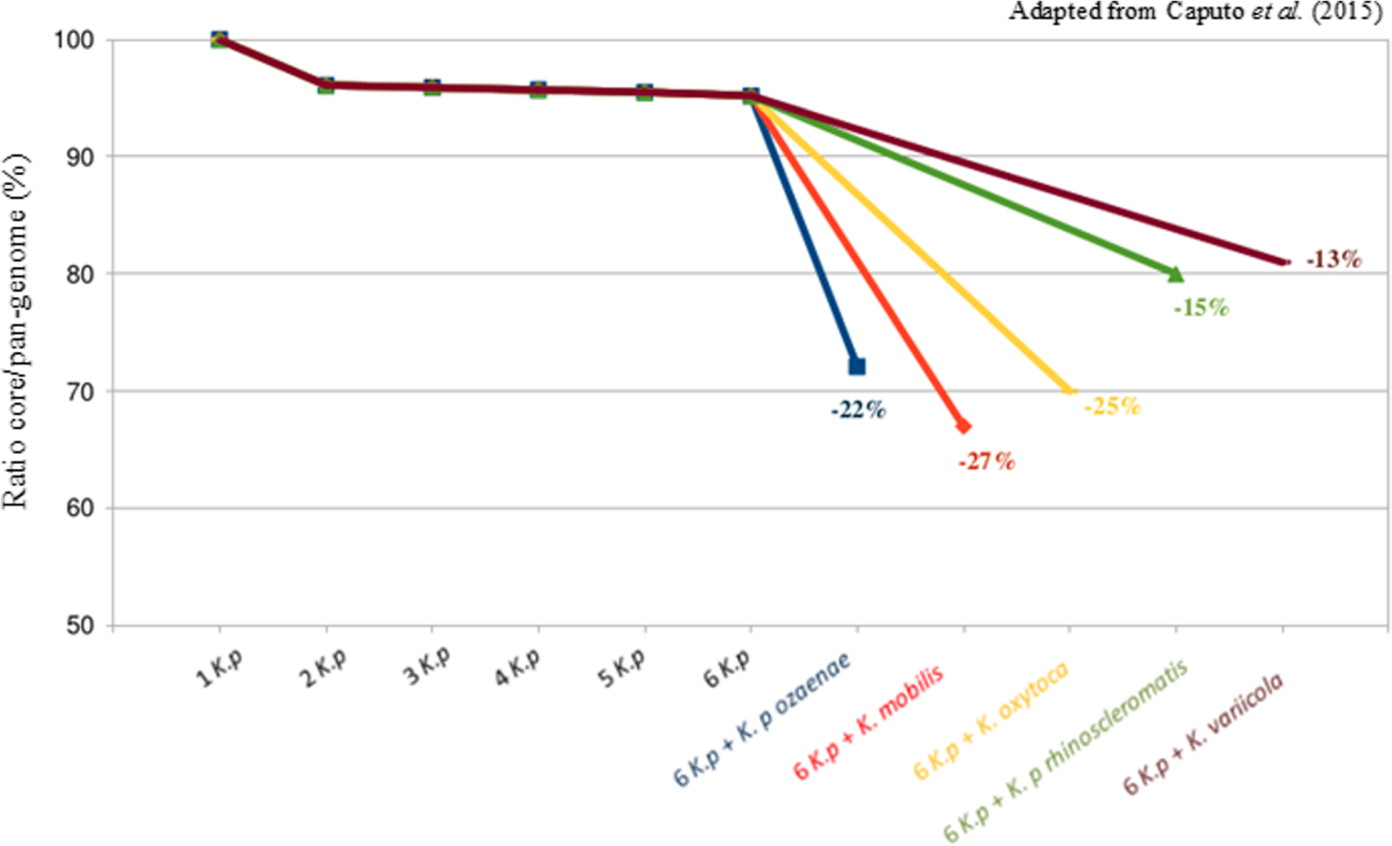
The core/pan-genome ratio for all strains of *Klebsiella* studied

PNan-genome analyses were performed for different strains and subspecies of *K. pneumoniae*, *K. oxytoca*, *K. variicola and K. mobilis* [36]. We determined that the core/pan-genome ratio for six *K. pneumoniae* subsp. *pneumoniae* strains was 94%. Then, we determined this ratio by comparing successively *K. pneumoniae* subsp. *pneumoniae* to *K. mobilis*, *K. variicola*, *K. oxytoca*, *K. pneumoniae* subsp. *ozaenae* or *K. pneumoniae* subsp. *rhinoscleromatis* genomes (Fig. 3). The ratios obtained were 67, 81, 69, 72 and 79% respectively. Therefore, we observed a discontinuity variation in the ratio for each of these species/subspecies, with a difference ranging from 13 to 27% with the bona fide *Klebsiella pneumoniae* species. We estimate that this ratio break is greater than 10% with no transition zone and reflects individual biological species. Accordingly, the authors said that *K. pneumoniae* subsp. *ozaenae* or *K. pneumoniae* subsp. *rhinoscleromatis* can be considered as species. A recent study has demonstrated the same break in the core/pan-genome ratio of *E. coli* strains, after the additions of *Shigella* strains [122]. The COG analysis and the KEGG analysis showed large differences which once again highlighted the very distinct genomic content of *K. pneumoniae* subsp. *ozaenae* or *K. pneumoniae* subsp. *rhinoscleromatis.*

This pan-genomic analysis enabled to conlude that *K. pneumoniae* subsp. *ozaenae* or *K. pneumoniae* subsp. *rhinoscleromatis*, which exhibit as many differences between them as with other *Klebsiella* species, may be distinct *Klebsiella* species [36] or, following Ereshefsky’s eliminative pluralism on species philosophy [123], distinct pan-genome-derived species.

## Conclusions

Since the introduction of DNA sequencing by Sanger and Coulson in 1977, great progress has been made. A growing amount of data is being generated, requiring continuously advanced computer processing. Numerous studies illustrating different methods have been published in different fields, such as genome assembly and annotation, as well as research on new bacterial species and the taxonomic classification of bacteria.

Regarding genome analysis, a complete genome of *Akkermansia muciniphila* was obtained directly from human stool samples by an original approach. Based on taxonogenomics, the creation of *Microvirga massiliensis* sp. nov. containing the strain JC119^T^ could be done.

Taxonomy traditionally operates with the principle of discontinuous variation. A break in the ratio of the core/pan-genome means that there is no transition from one species to another, leading to a definition of different species. This leap in the ratio represents a major difference between genomes. These irreconcilable differences cannot exist within a single species. The great discontinuity variation in the core/pan-genome ratio observed in *Klebsiella* species may help to redefine these species. Thus, we believe that pan-genome studies can help to better visualize gene content differences, inform species (re)definitions or classify species on their own according to discontinuous genomic content. However, this strategy will have to be empirically and systematically tested in the currently proposed genera.

Genomics challenges taxonomy. We are at the very beginning of the interpretation of the genome for taxonomic purposes.

## Abbreviations

ANI: Average nucleotide identity
ARG: Antibiotic resistance gene
BSR: Blast score ratio
COG: Cluster of orthologous group
DDH: DNA-DNA hybridization
GGDC: Genome-to-genome distance calculator
GOLD: Genomes online database
ITS: Internal transcribed spacer
KEGG: Kyoto encyclopedia of genes and genomes
LPSN: List of prokaryotic names with standing in nomenclature
MALDI-TOF: Matrix-assisted laser desorption/ionization time-of-flight
MLSA: Multilocus sequence analysis
NGS: Next-generation sequencing
SCS: Single-cell sequencing
SNP: Single nucleotide polymorphism
WGS: Whole-genome sequencing
